# Faith Healers and the Law

**Published:** 1903-11

**Authors:** 


### Faith Healers and the Law.

THE unrighteous cause of faith cure and similar hocus pocus has received rather a severe setback in this state, through a recent decision of the Court of Appeals, which legally established the criminality of parents or guardians who refuse or neglect to provide proper medical attendance for children in their care. The opinion is important, because it firmly defines the term "medical attendance," and will have an important bearing on all cases which may hereafter come up involving that question. And now that such a decision has been made it is quite probable that the District Attorney of Erie county may be more successful in his prosecution of the fakes and fakirs who are brought to his attention.

The case on which the decision was made was that of Luther Pierson who, on March 21, 1901, was arrested at White Plains for failing to provide medical attendance for his two-year-old daughter, who was ill of pneumonia, and who died on February 15. On May 23, he was found guilty of a misdemeanor and sentenced to pay a fine of $500, or spend 500 days in prison. His attorney filed notice of appeal, and two days later Pierson was released on bail. The case was taken to the appellate division, and Pierson made the statement that he was a divine healer. He was asked if this was not the same as christian science, and replied, "No; divine healing is diametrically opposed to the diabolical counterfeits, which are utterly anti-christian. These impostures are only seductive forms of spiritualism."

When the case was considered by the appellate division Justice Bartlett, who wrote the opinion reversing the conviction, held that the "medical attendance" referred to in the statute does not mean exclusively the attendance of a medical practitioner in the general sense of the term. It is worthy of note that Justice Goodrich dissented from this opinion.

An appeal was then taken to the Court of Appeals by District Attorney Andrews, the prosecuting officer, and after careful consideration of the merits of the case Justice Haight wrote an opinion reversing the appellate division and sustaining the trial court. The opinion declares dependence on faith in healing in a case of sickness to be criminal negligence. In delivering the opinion of the court, Justice Haight said:

It would seem that the legislative intent is reasonably clear, although possibly more precise language could have been employed. The section of the code under which the indictment was found contemplates that there are persons upon whom the law casts a duty of caring for minors. We are aware that there are people who believe that the Divine power may be invoked to heal the sick, and that faith is all that is required. There are others who believe that the Creator has supplied the earth, nature's storehouse, with everything that man may want for his support and maintenance, including the restoration and preservation of his health, and that he is left to work out his own salvation, under fixed natural laws. There are still others who believe that Christianity and science go hand in hand, both proceeding from the Creator; that science is but the agent of the Almighty, through which he accomplishes results, and that both, science and Divine power may be invoked together to restore diseased and suffering humanity. But, sitting as a court of law for the purpose of construing and determining the meaning of statutes, we have nothing to do with variances in religious belief, and have no power to determine which is correct. We place no limitations upon the power of the mind over the body; the power of faith to dispel disease, or the power of the Supreme Being to heal the sick. We merely declare the law as given us by the legislature. We find no error on the part of the trial court that called for a reversal.

Since the handing down of the opinion, John Alexander Dowie, of Zion, Ill., of whose Christian Catholic Church Pierson was a member, has entered New York on a crusade of purification. A reasonable time had been given Pierson to pay his fine, and not having done so, the district attorney applied for a bench warrant for his arrest. As it was being made out Pierson's attorney entered the court and paid the fine. Almost immediately after that the attorney, who defended the faith healer, secured an attachment against Dowie for something over $1,000, for fees in connection with the Pierson case, claiming that he was retained at Dowie's request. In the meantime Dowie had folded his tents and slipped away from New York, the scene of one of the most miserable fiascos in the way of a religious crusade the world ever saw.

The opinion stands and it applies to faith curists, faith healers, christian scientists, magnetists, vibrilists, and all the rest of the fakes and fakirs.

---

The increase of tetanus, resulting from Fourth of July injuries, is attracting attention all over the country. Medical societies and medical journals are discussing means and methods of controlling this horrible disease and its ghastly ravages. Important action on this subject was taken by the Mississippi Valley Medical Association during its recent meeting at Memphis. The secretary, Dr. Henry Enos Tuley, of Louisville, transmits to us a summary of the proceedings on that topic, with the request for publication of the same. We gladly comply therewith and invite the attention of our readers to a serious consideration of the subject. It will require an active campaign on the part of physicians and educators to convince the small boy of the heathenish course he is pursuing in the present methods of celebrating independence day. There are also some larger boys that need instruction on this point and, perhaps, some parents might be included.

Here is the report of the society referred to:

In view of the fact that more than 400 deaths from tetanus occurred following the Fourth of July celebration of 1903, as shown by the statistical report elaborated by Dr. S. C. Stanton, of Chicago, and published in the *Journal of the American Medical Association* of August 29, 1903, the great majority of which might have been prevented had proper precautions been taken; therefore

*Be it Resolved,* That the conclusions which follow, as offered by Dr. Stanton in a paper presented before the association, at the Memphis meeting, be endorsed as the sense of this association, and further

*Be it Resolved,* That the secretary be instructed to forward a copy of these resolutions and conclusions to the medical press, associated press, and the secretaries of the several state medical societies, with the request that they publish same and take suitable action thereon.

1. Enforcement of existing laws regarding the sale of toy pistols and other dangerous toys.
2. Enactment of laws by the nation, states and municipalities prohibiting the manufacture and sale of toy pistols, blank cartridges, dynamite canes and caps, cannon crackers, etc.
3. Open treatment of all wounds, however insignificant, in which from the nature or environment there is any risk of tetanus.
4. Immediate use of tetanus antitoxin in all cases of Fourth-of-July wounds, or wounds received in barnyards, gardens, or other places where tetanus infection is likely to occur.
5. As a forforn hope, the injection of tetanus antitoxin after tetanus symptoms have appeared.

---

The importance of a knowledge of the history of medicine has been over and again asserted in the columns of medical journals within the last few years. The profession is becoming roused to the propriety of establishing chairs in the medical schools for the purpose of teaching this hitherto much neglected topic. We are pleased to learn that the University of Maryland has taken the initiative in establishing such a professorship to which Dr. Eugene F. Cordell, of Baltimore, has been appointed. Dr. Cordell's essay on the Doctors and Medicine of Horace, published lately in *Johns Hopkins Hospital Bulletin,* has attracted much attention, and we may expect to see more from his pen of a similar nature. We trust other medical schools will follow the example of the University referred to.

THE fashion adopted some time ago by the *New York Medical Journal* of publishing signed editorials, is not likely to become popular with medical journals. It destroys the impersonality of editorial contributions and leads to unwise complications in editorial discussions. Moreover, it is doubtful whether such a method could be pursued for any considerable length of time without exhausting the field of editorial writers. On the whole, we do not believe the new fashion will become the prevailing mode among medical periodicals.

